# A call to ban the sale of tobacco products

**DOI:** 10.3389/fpubh.2022.904971

**Published:** 2022-11-10

**Authors:** Zhaohui Su, Dean McDonnell, Ali Cheshmehzangi, Junaid Ahmad, Sabina Šegalo, Claudimar Pereira da Veiga

**Affiliations:** ^1^School of Public Health, Southeast University, Nanjing, China; ^2^Department of Humanities, Institute of Technology Carlow, Carlow, Ireland; ^3^Faculty of Science and Engineering, University of Nottingham Ningbo China, Ningbo, China; ^4^Network for Education and Research on Peace and Sustainability, Hiroshima University, Hiroshima, Japan; ^5^Rufaidah Nursing College, Peshawar, Pakistan; ^6^Faculty of Health Studies, University of Sarajevo, Sarajevo, Bosnia and Herzegovina; ^7^Marketing, Fundação Dom Cabral—FDC, Nova Lima, MG, Brazil

**Keywords:** tobacco industry, ban, cancer, public health, health policy, intervention

## Abstract

Tobacco is both toxic and addictive. Mounting evidence shows that tobacco use has a detrimental impact on almost every aspect of human health, causing or worsening deadly public health crises from the cancer epidemic to the COVID-19 pandemic. However, while tobacco use is a threat to both personal and public health, it continues to surge across the world, especially in China and other low- and middle-income countries. To this end, this article argues in favor of using a ban on the sale of all tobacco products as a practical solution to the global tobacco use epidemic. It is our hope that insights provided by our work will inspire swift policy actions in countries such as China and beyond to curb the tide of rising tobacco consumption, so that populations around the world could be better shielded from the pervasive and long-lasting damage that tobacco products cause or compound.

## Introduction

Tobacco is toxic. A preponderance of evidence shows that tobacco smoking has a detrimental impact on almost every aspect of human health, causing or worsening deadly epidemics from cancer to coronavirus disease 2019 (COVID-19) (1). Yet, despite the growing body of evidence that reinforces and restates its damaging impacts on personal and public health (2–7), tobacco use remains prevalent across the globe. Analyses, for instance, showed that, in 2019, there were 1.14 billion people who were current smokers; throughout a period of nearly three decades, they consumed more than 7.4 trillion cigarette equivalents of tobacco products (8). This cumulative consumption has exerted a sobering toll on society. Approximately 8 million lives have been lost to tobacco-related diseases each year (9), making the global death toll from tobacco use greater than the mortality of AIDS, malaria, and tuberculosis combined. Tobacco use is also highly addictive. In a 2018–2019 analysis of 87,709 participants aged 20–69 in China, researchers found that against the backdrop of an already high prevalence of 25.1% of current smoking, men in China had an even higher rate−47.6% (10). These sobering statistics help explain why once people initiate and become accustomed to tobacco use, the addiction becomes entrenched and exceedingly difficult to manage, even when there is access to evidence-based interventions that are easy to adopt, such as technology-based programs (11).

Taken together, these revelations explain why tobacco use exerts such a heavy burden on society—it costs the global economy $1.4 trillion each year, ranging from expenses incurred from healthcare utilization, lost productivity, fire damage, to cigarette litter-induced environmental harm (12). Unfortunately, these alarming data and trends are not subsiding, underscoring the growing need for more effective interventions to curb tobacco use around the world, especially in low- and middle-income countries—where over 80% of tobacco users live, individuals who often lack access to health care infrastructure that is essential to effectively treat and manage their addiction (1).

Although global tobacco control efforts have been ongoing for quite some time (13–21), they are often too fragmented to comprehensively address the tobacco use epidemic in a fundamental fashion—people's exposure and easy access to tobacco products. This means that, rather than incentivizing the tobacco industry to transform its businesses into those that focus on health-promoting goods or services, existing tobacco control policies often contain too many loopholes that allow these companies to circumvent accountability for their products' negative health impacts. Ranging from surreptitious marketing practices to the wide dissemination of addictive e-cigarettes (22–24), the industry has continued to perpetuate the global tobacco epidemic rather than to temper it. Even the recent tobacco control policy developed by the United States (U.S.), which aims to ban menthol-flavored cigarettes and related products (25), may lead to mixed results or unintended consequences. Announced in April 2022 and considered a public health win, the U.S. Food and Drug Administration's draft rule may be too narrow to limit or discourage people's access to tobacco products (26). A 2022 study that gauges current American smokers' responses to the forthcoming ban shows that 51% of the participants, who were recruited online anonymously, said they would use non-flavored cigarettes as alternatives (27).

Policies that can be bypassed by alternative tobacco products (e.g., e-cigarettes) could also inadvertently promote tobacco use and pose unnecessary barriers to people who are trying to quit. Analyzing findings of three randomized controlled trials that involve 1,607 smokers, for instance, researchers found that, compared to gradual cessation, abrupt smoking cessation is more likely to yield positive outcomes (28). This research suggests that a number of ongoing factors continue to counter the years of interventions implemented to reduce tobacco use. The fact that tobacco consumption remains a global epidemic around the world, underscores the urgent need for further tobacco control actions especially policies that are more comprehensive and could more directly reduce the tobacco industry's ability to expand public access and exposure to tobacco products. To this end, this article explores the advantages and potential trade-offs of a ban on the sale of all tobacco products as a practical policy intervention for combatting the global tobacco epidemic. Using China as an example, we argue that such a ban in a country with the world's largest population is (1) reasonable, (2) feasible, and (3) can benefit the broader global community. Any change, however small, in China (given the size of its population—around 1.5 billion people—and the scope of its economy—second-largest globally in terms of its gross domestic product) can profoundly help move the needle forward to stem the tide of tobacco use worldwide.

## Secondhand smoke: An unintended consequence of using tobacco products

A tobacco product could be understood as “a product that can be consumed and consists, even partly, of tobacco” (29). In the context of this article, tobacco products refer to both conventional (e.g., cigarettes) and newer iterations (e.g., e-cigarettes). Unlike nutritious foods and effective medicines, which are essential for maintaining and sustaining personal and public health, tobacco products are deleterious and dispensable. Different from substances that are beneficial to human health and productivity, such as tea and coffee (30–32), almost all aspects of tobacco consumption are detrimental to personal and public health (33). Tobacco's damaging impacts on global health centers on its unique mode of consumption. Different from other addictive substances like cocaine, the smoke generated by tobacco products not only harms the health of the direct users (i.e., smokers), but also that of people who happen to be in the vicinity of the secondhand smoke.

In an analysis of data from 1990 to 2016, researchers found that even though progress has been made, the consequences of tobacco use on non-smokers remain high—i.e., in 2016, for every group of 52.3 individuals who smoked a mean of 24 years, there was an associated death of 1 individual attributable to secondhand smoke exposure (34). Though most, if not all, governments across the world agree on the end goal when it comes to tobacco control—elimination or eradication of tobacco use across society, especially among the young and vulnerable, they differ drastically in terms of the measures they implement to regulate tobacco use (33). One important example is China—the country is struggling with the damage that tobacco use has already caused but yet has the opportunity to make meaningful impacts by adopting and implementing a more coordinated tobacco control effort *via* a ban on tobacco products.

## Case example: Impact of tobacco use in China

When it comes to tobacco products, China is simultaneously the world's largest producer, the biggest consumer, and its most traumatized victim (35). In a study of 71 countries—accounting for over 95% of the world's total cigarette use and 85% of the global population—between 1970 and 2015, researchers found that not only has China's tobacco use been skyrocketing, but its total consumption of cigarettes, in 2013 alone, was 2.5 million metric tons (MMT); this was greater than the combined consumption of the next 39 highest countries during the same period, including Russia (0.36 MMT), the U.S. (0.28 MMT), and Japan (0.20MMT) (36). Comparatively, China bears arguably the most alarming toll of tobacco use worldwide. It is estimated that approximately 4,100 people per day in China died from tobacco-related diseases (estimate from 2022) (37). This sobering statistic is projected to jump to 6,000 deaths per day−3 million per year—by 2050, if effective interventions are not taken or remain a low priority in the country (37). The high prevalence of tobacco use in China is even more chilling if the negative impacts of secondhand smoke exposure are considered. The World Health Organization reports that over 700 million non-smokers in China are exposed to secondhand smoke, among which, 180 million are children (35). Yet despite the sobering toll of tobacco use, the evidence of which has been accumulating for decades (38–41), China has been lagging behind in developing and adopting impactful policies and other effective tobacco control interventions to fight the growing health and socioeconomic repercussions caused by this adverse behavior (42).

## More stringent tobacco control policies and related interventions are needed

**Figure 1:**
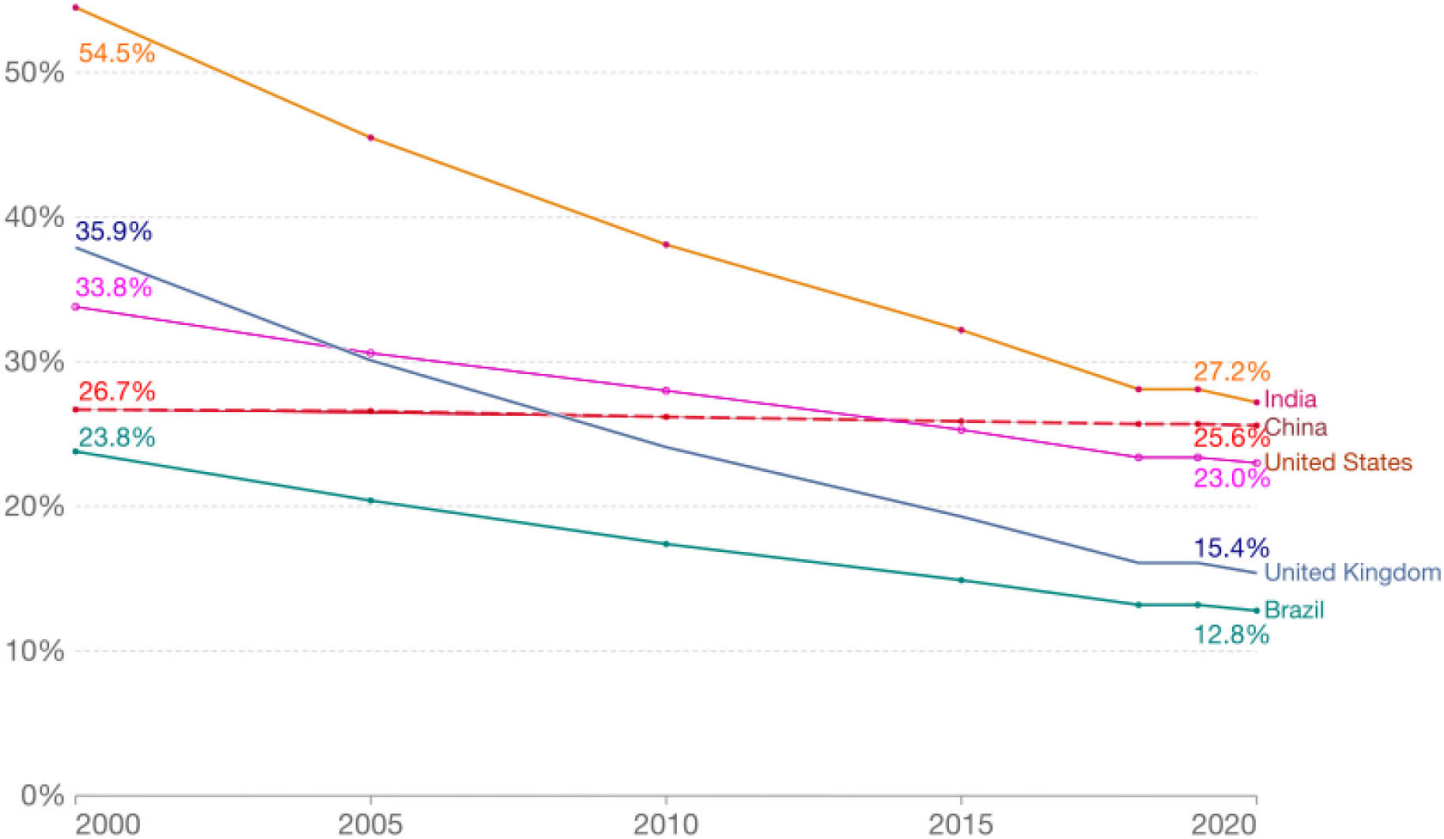
Percent of adult smokers from each of the selected countries, 2000 and 2020. Data source: The World Health Organization; Adults: people aged 15 years and older.

As illustrated in Table 1, meaningful tobacco control policies, especially those that can promote material changes in tobacco-related morbidity and mortality rates in the population have historically been negligible in countries like China. By contrast, tobacco control efforts have been more robust in developed nations such as the U.S. In spite of highly visible home to household tobacco brands such as Marlboro and powerful tobacco industry advertising, which normalized and socialized smoking with the zeitgeist at the time—from personal liberty to women's rights (75–77), the U.S. has been able to gradually and substantively reduce tobacco use among its diverse populations during the past several decades (78). Between 2000 and 2020, adult smoking rates in the U.S. dropped from 33.8 to 23.0% (78). Similar achievements have been observed in other countries such as India, the United Kingdom and Brazil. China, as one of the largest countries in the world, did not share in these improvements (see Figure 1) (78), though the scale, scope, and severity of its tobacco epidemic are simply too glaring to ignore, especially when they are coupled with known shortcomings of moderate control policies. These insights, collectively, suggest that stronger and more straightforward legislative actions, such as a ban on the sale of all tobacco products across the entire country (79–82), are urgently needed to avoid the catastrophic health and socioeconomic consequences that will likely mushroom as a result of the country's ever-growing tobacco use epidemic.

**Table 1:** Historical timeline: Key factors that shape tobacco use in the U.S. and China.

**Year**	**Key factors that shape tobacco use in the U.S. and China (highlighted)**
1954	The link between smoking and lung cancer was definitively established by Drs. Richard Doll and A. Bradford Hill (43)
1964	The U.S. Surgeon General's report officially linked smoking with deadly health conditions such as heart disease and lung cancer (44)
1966	Health warnings—“Caution—cigarette smoking may be hazardous to your health”—first appeared on cigarette packages sold in the U.S. Subsequent research investigations reveal conflicting effects when it comes to their impacts on smoking cessation (45–47)
1975	The first law that mandates designated smoking areas in public spaces, the Minnesota Clean Indoor Air Act, went into effect. This law, along with subsequent location-specific limitations on smoking, fuelled some of the earliest global research on tobacco control policies (48)
1984	Nicotine gum becomes the first modern drug to facilitate smoking cessation. It is also one of the most studied “alternatives” to tobacco use under the Nicotine Replacement Theory (49)
1982	The state-owned tobacco company, China National Tobacco Corporation, was founded. The Corporation is currently one of the most profitable companies in China in terms of profit (50). In 2021, the total tax and profit of the China National Tobacco Corporation is around 1,244.2 billion RMB (US $ 185.7 billion)— a 6.08% rise from 2020 and greater than Apple's global profits during the same period
1986	The harmful effects of secondhand second were officially acknowledged in the 19th U.S. Surgeon General report (51)
1987	Aspen, a U.S. city in the State of Colorado, becomes the first city to mandate smoke-free restaurants. In the same year, Joe Camel—a cartoon character that was created by tobacco company RJ Reynolds exposed millions of children to tobacco products (52). Cartoon as a persuasion technique has also been explored by e-cigarette makers (53)
1988	Proposition 99, which mandates an increase of cigarette tax by 25 cents in California, becomes the momentum that prompts the U.S. to utilize tax revenues from tobacco products for smoking cessation efforts (54)
1990	San Luis Obispo, a city in California, becomes the first city in the world to ban smoking in all public buildings (55)
1994	A cohort of 7 tobacco company executives testifies under oath in front of U.S. Congress, indicating that they do not believe nicotine—what propels tobacco use's addictiveness—is not addictive (56)
2003	China joined the World Health Organization Framework Convention on Tobacco Control (FCTC). It is estimated that around 12.8 million smoking-related deaths could be prevented from 2013 to 2050 in China alone, if the FCTC guidelines are fully implemented (57)—an ideal that has yet to be achieved in the country as of 2022
2006	The final ruling of the U.S. Department of Justice's 1999 suit against the tobacco industry for a “coordinated campaign of fraud and deceit,” among other things (58), has concluded that the industry has lied to the public about the harms of tobacco use for over half a century. In the same year, e-cigarettes were first introduced in the U.S. (59). Though viewed as promising by some, e-cigarettes' utility and functionality as mechanisms that could facilitate smoking secession are, at best, mixed (60–62)
2009	China increased its tax on tobacco products by 11.7% at the producer price level. The effects, though, are minimal at best: (1) unchanged specific excise tax: remain to be 0.06 RMB (US $0.0090) per pack; (2) a 3.4%-point increase in the retail price tax rates to boost the cigarette tax from 40 to 43.4% (63), considerably lower than the FCTC recommended level of 75%
2010	Tobacco marketing is prohibited from using potentially misleading descriptions such as “low” or “light”, which could create false beliefs about smoking risks (64), to persuade vulnerable populations, particularly youths, into (continued) smoking
2014	China's State Council issued a draft regulation on “Smoking Control in Public Places” in November 2014, which proposes a ban on smoking in most public spaces (e.g., workplaces and public transport) as well as a ban on tobacco advertising. Though much of the regulation has already been proposed by the Ministry of Health in 2011 and the bans are widely considered long overdue (65), the regulation only came into effect in June 2015
2015	Tobacco advertising to minors, in public places and transportation, as well as in outdoor areas is prohibited per China's revised national Advertising Law that went into effect in 2015. Yet recurring evidence shows poor compliance across society (66–68). Not to mention that online tobacco marketing in shared “public” spaces such as social media is complex to monitor or regulate and has been thriving in China (69)
2019	The “Tobacco 21” law mandates all states in the U.S. to prohibit the sale of tobacco products to people aged under 21 years (70)
2020	All e-cigarette manufacturers are required to submit to the U.S. Food and Drug Administration (FDA) a premarket review application to ensure their products do not pose as a threat to public health
2021	The U.S. FDA proposes a ban on menthol-flavored cigarettes (25)
2022	By denying further marketing authorization, the U.S. FDA effectively banned the sale of Juul e-cigarettes in June 2022 (71), one of the most popular brands among e-cigarette smokers, most of whom are children and young adults (72, 73)
2023	Due to countersuits filed by the tobacco company, a graphic warning mandate on cigarette packages—a communication mechanism that could meaningfully promote smoking cessation (74)—proposed by the FDA will be postponed to 2023, if not later

## A ban on the sale of tobacco products

A ban on the sale of tobacco products is the prohibition of the sale or purchase of tobacco products, including e-cigarettes, across sectors of society within its borders. Again, using China as an example, this means that all business-to-business or person-to-person exchanges of tobacco products will be prohibited in the country, including international e-commerce. Different from a comprehensive ban on all tobacco-related activities, like the one adopted in Bhutan (83), a ban on the sale of tobacco products would only prohibit the exchange of tobacco products—both legally or in the back market within the borders of China, as opposed to controlling people's rights to access or use of tobacco products, not the least of which because the latter raised legal issues and could be extremely difficult to monitor or control. Under a sales ban, residents in China could still make international trips to obtain tobacco products, or for those who are truly determined, grow their own tobacco leaves, but they are not allowed to exchange these products. Similarly, tobacco farmers and corporations could still grow or manufacture tobacco products, but these products would not be allowed to be sold to people living in China. Compared to existing strategies, this policy intervention bears a multitude of advantages, most of which are its feasibility and practicality, not to mention a strong first step forward in addressing China's tobacco use epidemic.

## Feasibility of establishing the ban

There are a number of reasons to call for action on a ban on the sale of all tobacco products. First, such a ban is simple and straightforward to understand and to implement. A common pitfall of many existing tobacco regulations is that they are often too complicated for the general public to understand or appreciate (84). A ban on the sale of all tobacco products means that tobacco products will no longer be available in the marketplace, or exchanged between people or businesses—a relatively straightforward policy that can be understood by tobacco users across age, education, or other socio-demographic spectra, as well as by other stakeholders such as sellers, marketers, and law enforcement agencies. A ban on the sale of all tobacco products is less complex than nuanced bans that are often seen in developed countries. A straightforward ban would also be more in line with many developing countries' public health realities. Different from high-income nations like the U.S., which have been changing the public's attitudes toward tobacco consumption for decades, countries like China, which have a more pronounced prevalence of smoking and less available public health resources, may need more rigorous and less reserved interventions to prevent their tobacco use epidemic from further expanding in a timely manner. A ban on the sale of all tobacco products, regardless of their flavors or modes of consumption, would be much easier to follow and carry out under these countries' circumstances.

Second, the people-first focus of the proposed ban could help facilitate public adherence. As opposed to prioritizing politics or profits, a ban on the sale of all tobacco products validates the government's determination and devotion to protecting public health, above and beyond short-term considerations such as political gains or losses. Considering the Chinese government's recent response to the COVID-19 pandemic, using a whole-of-society zero-COVID strategy approach, a direct and people-first policy from the government is not unattainable and could help the public better understand and appreciate the severity of the global tobacco use epidemic and adjust their mindset accordingly to comply with the policy. Third, similar to other countries that are burdened with the tobacco use epidemic, China has the urgency and the capacity to carry out a ban on the sale of all tobacco products successfully. Presently, approximately one in every three global tobacco users lives in China (9)—populations that are likely to both personify and perpetuate the country's raging double-whammy epidemics—the tobacco use epidemic and the cancer epidemic (85).

Fourth, in addition to tobacco-related morbidity and mortality, China's economic health is considerably compromised by the tobacco use epidemic. It is estimated that, in 2017 alone, the economic tolls of lung cancer on the country have reached over $25 billion (86), a considerable amount of financial burden that a ban on the sale of tobacco products could help in lowering. As such, China has the administrative motivation to carry out whole-of-society policies like a ban on the sale of all tobacco products across society. Fifth, and perhaps most importantly, China has the agency to ban the sale of tobacco products. For starters, the world's largest producer of cigarettes, China National Tobacco Corporation, is state-owned. On one hand, the nature of the company reveals how entrenched the tobacco industry is in the administrative fabric of China (87). But on the other hand, this also means that as long as officials in China are willing and committed, they could effectively implement a ban on the sale of all tobacco products without the need of back-and-forth negotiations with private sectors, as seen in other countries (88).

The largely state-owned nature of China's tobacco industry means that, when political will is well-established, government officials can instruct and transform its existing tobacco industry workforce into other industries that do not produce products that are debilitating to national and global health. Amid the COVID-19 pandemic, to protect children and adolescents from becoming too addicted to online gaming, China successfully regulated the duration of which youths could be exposed to these entertainment venues (89). In a similar vein, to alleviate the burden on school students and their parents, in 2021, China also banned for-profit private tutoring across the country (90). Taken together, these recent events and actions by China suggest that the world's largest country (by population size) has the capability to ban the sale of all tobacco products within its borders. It would be impactful if this can be accomplished by China in a timely manner, since, substance use, which is highly associated with tobacco use, has been prevalent and is on the rise across the globe (91). By designing, developing, and delivering bans that could eradicate the public's vulnerability toward addictive substances like tobacco, China could serve as a harbinger in the protection of the health and quality of life of the global community, above and beyond those living within its borders.

## The broader implications and potential effects of banning tobacco products

The impacts of a ban on the sale of tobacco products could be largely categorized into two types: desirable outcomes and unintended consequences. A wide range of positive changes could be expected from the said ban. First, in addition to the welcoming impacts on society discussed earlier, a ban on the sale of tobacco products could also help countries across the globe better cope with the negative consequences of COVID-19 on personal and public health. Recurring evidence shows that COVID-19 could cause greater adverse health consequences to people with damaged lungs, such as tobacco users (3, 92, 93). A review of evidence on 32,849 COVID-19 patients across the globe shows that people with any smoking history experienced significantly more severe COVID-19 symptoms and worse hospitalization outcomes compared to non-users (92). In a study of 6,003 Italian adults amid the pandemic, researchers found that the total tobacco consumption has further increased by 9.1% (94). These findings suggest that there may be a vicious cycle between tobacco use and heightened COVID-19 risks. As COVID-19 continues evolving (95–99), and, paired with the rising presence of infectious diseases and geopolitical conflicts that could be equally damaging to our health systems (100–103), a ban on the sale of tobacco products should also be seen as an even more necessary step, since the vicious cycle between tobacco use and COVID-19 infections has been shown to worsen health conditions and lead to severe COVID-19 disease and death.

Second, a reduction in tobacco use could also persuade tobacco growers and producers to switch to other products or industries that do not harm planetary health. If history is a sagacious guide, the ultimate market force—synergistic dynamics between supply and demand—could be the best shadow policymaker for phasing out the tobacco industry. A ban on tobacco sale (demand drops) could lead to a material reduction in the marketability of tobacco products (short-term loss of profitability), which in turn, has the potential to incentivize tobacco farmers and producers to grow and market health-promoting crops instead of tobacco (long-term supply chain transformation). This would, effectively leverage the market forces and strategically use them to promote positive societal changes. Considering Chinese consumers' growing purchasing power and subsequent sway in the global economy (China has the largest population in the world and a growing economy), and the fact that China's largely state-owned tobacco industry is closely connected with the global tobacco scene (50, 104), the said ban, when optimally executed, has the potential to reduce the global presence of tobacco products right away in terms of sales and usage—the ultimate goal we hope tobacco control policies could achieve.

Third, it is also important to note that a ban on the sale of all tobacco products has the potential to introduce unintended consequences, such as enabling or deepening illicit markets for tobacco products. However, the potential for inadvertent outcomes neither means that they could not be predicted nor prevented. Policymakers in China and elsewhere, for example, could collaborate with researchers in academia, practitioners in the tobacco industry, public relations professionals and other experts and stakeholders to ensure that these unintended consequences are properly mitigated. Essentially, all policy interventions can yield both wanted and unwanted outcomes. A ban that could have unintended results should hardly surprise policymakers. Rather than pouring valuable public resources into developing less effective tobacco control policies, we believe it is more sensible and practical to invest in decisive intervention mechanisms, like the proposed ban, as its strength of impact and benefits should likely outweigh its unintended consequences (if any).

## Conclusion

Tobacco is toxic and addictive. The preponderance of evidence on tobacco use's harm substantiates the call for a stronger, more straightforward policy intervention that bans the sale of all tobacco products, especially for developed countries that are lagging behind in tobacco control wins and for developing countries that have limited public health infrastructure or resources to launch multi-modal campaigns to counter the tobacco industry's aggressive sales and advertising of tobacco products. Using China as an example, this article presented key rationales for advocating the adoption and implementation of such a ban, suggesting that it is a practical policy intervention for the world stage. It is our hope that the insights provided in this perspective will inspire swift policy actions in curbing tobacco use across the globe. In places like China where the population is enormous and the health infrastructure is tenuous, even an incremental change in the prevalence of tobacco use could lead to significant improvements in tobacco-related healthcare utilization and costs, as well as salutary decreases in human suffering from predictable and preventable tobacco-related diseases and deaths. Time is ripe for society to control the tobacco epidemic with a bang.

## Data availability statement

The original contributions presented in the study are included in the article/supplementary material, further inquiries can be directed to the corresponding authors.

## Author contributions

ZS, DM, AC, JA, SŠ, and CV conceptualized this work, reviewed the literature, as well as drafted and edited the manuscript for intellectual content.

## Conflict of interest

The authors declare that the research was conducted in the absence of any commercial or financial relationships that could be construed as a potential conflict of interest.

## Publisher's note

All claims expressed in this article are solely those of the authors and do not necessarily represent those of their affiliated organizations, or those of the publisher, the editors and the reviewers. Any product that may be evaluated in this article, or claim that may be made by its manufacturer, is not guaranteed or endorsed by the publisher.

## Abbreviations

COVID-19: coronavirus disease 2019
MMT: million metric tons
U.S.: United States
FDA: U.S. Food and Drug Administration.
